# Prevalence of institutional delivery and associated factors in Dodota Woreda (district), Oromia regional state, Ethiopia

**DOI:** 10.1186/1742-4755-9-33

**Published:** 2012-12-15

**Authors:** Addis Alem Fikre, Meaza Demissie

**Affiliations:** 1 Clear Impact Consult (CIC), P.O.Box: 876, Addis Ababa, Ethiopia; 2 Addis Continental Institute of Public Health, Addis Ababa, Ethiopia

**Keywords:** Institutional delivery, Ethiopia

## Abstract

**Background:**

Giving birth in a medical institution under the care and supervision of trained health-care providers promotes child survival and reduces the risk of maternal mortality. According to Ethiopian Demographic and Health Survey (EDHS) 2005 and 2011, the proportion of women utilizing safe delivery service in the country in general and in Oromia region in particular is very low. About 30% of the eligible mothers received Ante Natal Care (ANC) service and only 8% of the mothers sought care for delivery in the region. The aim of this study is to determine the prevalence of institutional delivery and understand the factors associated with institutional delivery in Dodota, Woreda, Oromia Region.

**Methods:**

A community based cross sectional study that employed both quantitative and a supplementary qualitative method was conducted from Jan 10–30, 2011 in Dodota Woreda. Multi stage sampling method was used in selection of study participants and total of 506 women who gave birth in the last two years were interviewed. Qualitative data was collected through focus group discussions (FGDs). Data was entered and analyzed using EPI info 3.5.1 and SPSS version 16.0. Frequencies, binary and multiple logistic regression analysis were done, OR and 95% confidence interval were calculated.

**Results:**

Only 18.2% of the mothers gave birth to their last baby in health facilities. Urban residence, educational level of mothers, pregnancy related health problems, previous history of prolonged labour, and decision made by husbands or relatives showed significant positive association with utilization of institutional delivery services (P < 0.05). While ANC attendance during the index pregnancy did not show any association.

**Conclusion:**

Institutional Delivery is low. Increasing accessibility of the delivery services and educating husbands not only mothers appear very important factors in improving institutional delivery. Health education on the importance of institutional delivery should also address the general population. The quality and content of the ANC services need to be investigated.

## Introduction

In spite of the national and global efforts at reducing maternal morbidity and mortality through the safe motherhood initiative, there is no significant reduction in maternal morbidity and mortality in developing countries [1]. The United Nations Children’s’ Fund (UNICEF) estimates that yearly about 515,000 women die of pregnancy and childbirth complications. It is also estimated that 1600 women across the world die each day as a result of pregnancy and childbirth related problems and the greater proportion of these deaths occur in developing countries [2-7].

In Ethiopia, maternal mortality and morbidity levels are among the highest in the world. The maternal mortality ratio currently is 676 per 100,000 live births [8]. One explanation for this poor health outcome among women is unavailability and low use of the available modern health services by a sizable proportion of women in Ethiopia [8,9].

According to Ethiopian Demographic Heath Survey 2011 and 2005, the proportion of women utilizing safe delivery service in the country in general and in Oromia region in particular is very low [8,9]. Only about a quarter of the eligible mothers received ANC service and only 8% of the mothers delivered in health facilities in the region [8]. Therefore, the purpose of this study is to understand the determinants of institutional delivery in the studied Woreda.

## Methods

### Study area and period

This study was conducted in Dodota woreda (district) which is one of the 16 woredas in Arsi zone of Oromia region, Ethiopia. The woreda is located 125 KMs South East of Addis Ababa, the capital city of Ethiopia. The woreda is structured in to 15 kebeles (the lowest administrative unit (12 Rural and 3 Urban). According to the 2005 censes the total population of the woreda is 72,000. The health infrastructure in the woreda comprises of 2 health centers, 12 health posts, and 2 clinics in the woreda. With regard to man power there are 2 health officers, 22 clinical nurses, 3 lab technician, 2 sanitarian, 3 druggist, 2 health assistance and 24 health extension workers in the woreda. Data was collected from January 12–30, 2011.

### Study design and population and sampling

A community based cross sectional survey was the main method of data collection. Women who gave birth in the last two years in Dodota woreda were the study population. Total sample size of 506 was calculated using a formula for a single population proportion, assuming a design effect of two and a non-response rate of 10%.

Multi stage sampling was used in selecting the study subjects. Dodota woreda has 12 Rural and 3 Urban kebeles (kebele is the smallest administrative unit in Ethiopia), 5 kebeles from the Rural and one kebele from the urban were selected by simple random sampling method. The sample size was distributed to the six kebeles proportionate to the size of their population. At the kebele level, households were selected by systematic random sampling based on the sampling frame obtained from kebele administration. The sampling interval of the households in each kebele was determined by dividing the total number of households to the allocated sample size. If more than one eligible woman were encountered in the household, a lottery method was used to determine the woman to be interviewed. When no eligible woman was identified in the selected house hold, the next household located in the right direction was visited.

### Data collection

A structured questionnaire which was initially prepared in English and then translated to the local language (*Afan Oromo*) was used to collect data. Six health extension workers (HEW’s) who are working outside the actual study area were trained for two days by the principal investigator to interview mothers. A pretest was done prior to the survey in similar setup outside the study area

Health extension workers are ‘health cadres’, secondary school complete member of community who are recruited and trained by the Ministry of Health to work as community health workers and are involved in prevention and health promotion works. Each Health Extension worker has its own household and is assigned to educate and support the community in many health issues.

### Qualitative data

In order to have an in depth insight on the factors related to place of delivery, data was also collected using qualitative data collection methods. Three Focused Group Discussions (FGDs) were held with mothers in the woreda. Each FGD had 10 participants. Recruitment of participants was assisted by Health Extension workers of the kebeles. The participants were within the age range between 19–72 years and each FGD’s took 45 – 60 minutes. The FGDs were facilitated by principal investigator assisted by the one research assistant. Written notes were taken; in addition all the discussions were tape recorded.

### Data analysis

The quantitative data were entered in Epi Info Version 3.5.1 then transported to SPSS 16.0 for analysis. Data exploration was done to visualize the general feature of the data. After exploration, univariate, bivariate and multivariate analyses were performed step by step.

Univariate analysis was done to describe some important centeracteristics of study subjects. The data were expressed in percentages, graphs, means and standard deviations. Then bivariate analysis using logistic regression technique was done to see the crude association between the independent variables and the dependent variable. The strength of association between dependent variable and independent variables (covariates) was expressed in odds ratio (OR). Finally multivariate analysis using forward stepwise multiple logistic regression technique was done to evaluate independent effect of each variable on health care delivery by controlling the effect of others.

### Ethical considerations

Institutional ethical clearance was obtained from Haramaya University and the Addis Continental Institute of Public Health Institutional Review Board (IRB). Study subjects were informed about the purpose of the study, their right to refuse and to withdraw. Informed verbal consent was obtained from each subject before data collection. Confidentiality of the data was kept by avoiding personal identifier and data was kept by the PI in a safe place.

## Results

The overall response rate was 506 (100%). Of the total respondents, 275 (54.5%) were illiterate, 436 (86.2%) were from Oromo ethnic group, 395 (78.1%) were housewives, 263 (52%) were Orthodox Christians, 467 (92.3%) were married; 329 (65%) had an income range between 100–499 Birr (which is approximately 6–28 USD) per month and 182 (36%) were in the age group 25 – 29 years with a mean age of 28.04 years. About 429 (84.8%) reported they were within the age range of 15–19 during their first pregnancy, 292 (57.7%) had history of 2–5 Pregnancies (Table 1). Among the respondents, 34 (6.7%) had history of abortion, 25 (5%) still birth and 90 (17.8%) had an infant death. (Table 1).

**Table 1:** Socio demographic centeracteristics of respondents in Dodota Woreda, Ethiopia, Jan 2011

**centeracteristics of respondents**	**Frequency**	**Percent**
**Age**		
**Mean ± SD**	28.04 ± 5.8	
15 – 19	25	4.9
20 – 24	105	20.8
25 – 29	182	36
30 – 34	102	20.2
35 +	92	18.2
**Educational level**		
No Formal Education	275	54.3
Only read and write	54	10.7
Elementary school	133	26.3
Secondary school	41	8.1
12+	3	0.8
**Ethnicity**		
Amhara	56	11.1
Oromo	436	86.2
Others	14	2.8
**Occupation**		
House wife	395	78.1
Farmer	86	17
Others	25	4.9
**Religion**		
Muslim	230	45.5
Orthodox Christians	263	52
Catholic Christians	13	2.6
**Marital status**		
Married	467	92.3
Others	39	7.8
**Income/ Month**		
< 100 Birr	46	9.1
100 – 499 Birr	329	65
≥500 Birr	131	25.9

### Institutional delivery, ANC care and history of reproductive health problems

Out of the 506 respondents only 92(18.2%) gave birth to their last child at health facility where as 414(81.8%) gave birth for their last child at home. Four Hundred fourteen (82%) of them had ANC care. With regard to obstetric complications for the index pregnancy; 466 (92.1%) of respondents report no problems before and during time of labor. Regarding the duration of labor, 371(73.3%) of them responded their labor took less than half a day, 100(19.8%) one day or one night, 30(5.9%) more than one and half day. Most of the respondents 493(97.4%) delivered alive baby. Among those who were born alive 492(97.2) didn’t face any problem. (Table 2).

**Table 2:** Health Facility delivery and history of reproductive health problem of the responding mothers, Dodota Woreda, Ethiopia, January 2011

**Pregnancy related history**	**Frequency**	**Percentage**
**Last child place of Birth**		
Home	414	81.8
HF	92	18.2
**Problems faced during/before labor**		
Yes	40	7.9
No	466	92.1
**Duration of last Labor**		
Less than half a day	371	73.3
One day or one night	100	19.8
More than one and half day	30	5.9
ANC attendance in last Pregnancy		
Yes	415	82.0
No	91	18.0

### Factors related with institutional delivery

The factors that were found to be associated with institutional delivery were residential place, educational level of mothers, previous history of prolonged labour, and final decision made by husbands or relatives. Those living in urban set up, literate, influenced by their husband and relative and those who had prolonged labour were more likely to deliver in modern health care. ANC attendance does not show association with institutional delivery. However, it was only 18% of those who had ANC attendance who gave birth at health facility (Table 3).

**Table 3:** Factors influencing place of delivery in Dodota Woreda, Ethiopia, January 2011

**Socio demographic**	**Institutional delivery**		**Crude OR**	**Adjusted OR**
	**Yes# (%)**	**No**	**(95% CI)**	**(95% CI)**
**Residence**				
Urban	73(62.4%)	44	1	1
Rural	19 (5%)	370	**32.3(17.84, 58.50)**	**22.8(10.57,49.42)**
**Level of Schooling**				
Secondary & Higher	19(43%)	25	1	1
Primary education	47(35.3)	86	1.39(0.69,2.79)	1.13(0.38,3.33)
Only read and write	4 (7.4%)	50	**9.50(2.92,30.92)**	4.10(0.8,21.05)
Illiterate	22(8%)	253	**8.74(4.18,18.29)**	**3.94(1.3,11.99)**
**ANC**				
Yes	75 (18%)	340	1	1
No	17 (18.7)	74	0.98(0.52, 1.80)	0.35(0.21,1.52)
**Preg. Related Problems**				
No	80(17.2%)	386	1	1
Yes	12(40%)	28	**0.48(0.24,0.99)**	**0.11(0.04,0.33)**
**Length of Previous Labor**				
Less than Half a Day	52(14%)	319	1	1
One Day or One Night	28(28%)	72	**0.42(0.25,0.71)**	0.52(0.24,1.13)
More than One and Half Day	12(40%)	23	**0.31(0.15,0.67)**	**0.20(0.07,0.61)**
**Final Decision**				
Self	38(9.5%)	364	1	1
Husband and others	54(48%)	50	**0.10(0.06,0.17)**	**0.14(0.07,0.29)**

### Qualitative results

Three FGDs were conducted involving a total of 30 participants, 10 in each group, with an age range of 19–72 years old women and all having at least one child. Most of respondents mentioned that they preferred home as delivery place, and almost all that had given birth in recent years gave birth at home. The main reason they stated was transportation problem; if they want to take a woman to health facility the available means of transportation is “Garri” which is a cart pulled by horse/donkey which is not convenient to transport pregnant women.

"“ labor comes suddenly, health facilities are far the only choice of transport on those moments is the gari which is not comfortable to anyone leave alone to a woman in labor” FGD participant."

The other reasons given for not delivering at health facility were 1) most of the time the decision to the place of delivery is made by the husband or relatives, 2) there is a stigma attached to a woman who delivers at health care, she will be considered weak, and her family members do not want to be labeled as weak. Therefore having a child at home is considered as sign of strength.

## Discussion

This study is a community based study in randomly selected kebeles. It included both urban and rural kebeles. The survey was also supplemented by data gathered by qualitative methods The study shows that the prevalence of institutional delivery is 18.2% and educational level of mothers; residential place, previous prolonged labour, and final decision made by husbands or relatives were factors associated with institutional delivery. ANC attendance did not show an increase in institutional delivery.

This slight difference between this study and the previous ones (13.5 Vs 18.2%) may be due to the introduction of Health Extension Workers (HEWs) in Ethiopia who can assist delivery at health post level and the expansion of the health facilities currently compared to the time the other studies were conducted. Institutional delivery is influence by place of residence; women who live in rural areas are less likely to deliver in health facility. This finding is in line with other studies done in our country [8-11]. For instance, women residing in Addis-Ababa are about 40 times more likely to deliver in health care facility than rural women, while women from other urban areas are about five times more likely to receive assistance during delivery than rural women [10,11]. This indicates the difference in access especially in terms of physical distance which is important for service utilization. If health facilities are not in close proximity or in walking distance, rural mothers are less likely to afford transportation cost. In many instances even if they can afford to pay the transportation fare, the vehicle may not be available at the time they need it.

Women education is a strong predictor of place of delivery. Those women with no education are less likely to deliver in health institutions than literates. This has also been recorded in other studies done in Ethiopia, Tanzania, Burkina Faso and India [10-14]. As educated women are expected to have knowledge about the risks of home delivery they are more likely to deliver at health institution as compared with those with little or no knowledge.

Another strong factor for utilization of health care for delivery is the length of previous labour. As the length of labour prolonged in the previous delivery the woman prefers to deliver in health institution, the same is true when a woman had other difficulties in the previous labours. These findings have also been documented in similar studies in North Gondar (Northern Ethiopia) and in India [11,14], the fear facing the same problem forces them to take precaution.

ANC attendance which is believed to increase institutional delivery, in this study doesn’t show any association and does not seem to increase institutional delivery. About 82% of the mothers had ANC follow up; out of those who attended ANC it was only 18% that had institutional delivery. One major outcome of ANC should be increasing the utilization of safe delivery service and reducing maternal mortality related to child birth. This calls for exploring the quality of ANC programs and the role they are playing in improving delivery at health facilities.

Husbands and relatives decisions to the place of delivery has an increasing effect of utilizing health care delivery than decision made by the mother herself, this finding is consistent with the research done in North Gondar [11]. If women are encouraged by husbands and relatives they would also get financial and other social supports to go to health facility which would allow them to have health care assisted delivery.

Sudden onset of labour combined with absent transport facility or lack of money for transportation cost are very important factors contribution to low institutional delivery. Low decision making power of the women is also associated with lack of financial resources or income in the rural mothers which leaves them to the decision of their husbands or relatives, similar conditions are also observed in other regions of the country and other countries [8,11,13].

The social stigma of being considered as weak by the mother and the mother’s family members is one major obstacle for utilizing health care facility for delivery. Since social interactions are very close and important in Ethiopian society it is not an easy thing to ignore community members opinion and this may require continuous interventions to change.

## Conclusion and recommendation

This study has revealed institutional delivery by mothers in the area is very low. Place of residence, maternal education, and husband and relatives decision are found to be very important for institutional delivery. Births attended by skilled personnel is crucial in ensuring safe delivery of mothers and hence reducing maternal death, however the majority of our mothers still deliver at home without any skilled person assistance. Therefore, policy makers and health care planners need to recognize the factors hampering institutional delivery and work on improving the situation, otherwise with such high birth unattended by skilled personnel our maternal mortality will remain one of the highest. More effort should be given to improving accessibility of the service especially the physical accessibility. Educating mothers and improving men awareness are the other important areas of intervention to improve service utilization. Increasing awareness of husbands appears more important than the mothers themselves because it is the husbands who have the financial and the decision making power. It is surprising that ANC attendance did not show an increase in institutional delivery, this raises question in the quality of our ANC service and calls for investigating the quality and content of the ANC service. Finally community members should get regular education as there are some misconceptions on women’s delivering in health facility. The woreda health offices and other responsible bodies should make efforts to increase community based health education on the risks of delivery unattended by skilled personnel to the mothers and the community at large.

## Competing interests

Both authors’ declare that they have no competing interests.

## Authors’ contributions

Author 1. AF: Initiated the research, wrote the research proposal, conducted the research, did data entry and analysis and wrote the manuscript. Author 2. MD: Involved in the write up of the proposal, data analysis, and write up of the manuscript. Both authors read and approved the final manuscript.
